# A Preliminary Study Examining the Binding Capacity of *Akkermansia muciniphila* and *Desulfovibrio* spp., to Colonic Mucin in Health and Ulcerative Colitis

**DOI:** 10.1371/journal.pone.0135280

**Published:** 2015-10-22

**Authors:** Helen Earley, Grainne Lennon, Aine Balfe, Michelle Kilcoyne, Marguerite Clyne, Lokesh Joshi, Stephen Carrington, Sean T. Martin, J. Calvin Coffey, Desmond C. Winter, P. Ronan O’Connell

**Affiliations:** 1 School of Medicine and Medical Science, University College Dublin, Belfield, Dublin 4, Ireland; 2 Centre for Colorectal Disease, St Vincent’s University Hospital, Dublin 4, Ireland; 3 Glycoscience Group, National Centre for Biomedical Engineering Science, National University of Ireland, Galway, Ireland; 4 Microbiology, School of Natural Sciences, National University of Ireland, Galway, Ireland; 5 College of Life Sciences, School of Agriculture, Food Science and Veterinary Medicine, University College Dublin, Dublin 4, Ireland; 6 Graduate Entry Medical School, Limerick, Ireland; Inserm U995-Université de Lille, FRANCE

## Abstract

**Background:**

*Akkermansia muciniphila* and *Desulfovibrio* spp. are commensal microbes colonising the mucus gel layer of the colon. Both species have the capacity to utilise colonic mucin as a substrate. *A*. *muciniphila* degrades colonic mucin, while *Desulfovibrio* spp. metabolise the sulfate moiety of sulfated mucins. Altered abundances of these microorganisms have been reported in ulcerative colitis (UC). However their capacity to bind to human colonic mucin, and whether this binding capacity is affected by changes in mucin associated with UC, remain to be defined.

**Methods:**

Mucin was isolated from resected colon from control patients undergoing resection for colonic cancer (n = 7) and patients undergoing resection for UC (n = 5). Isolated mucin was purified and printed onto mucin microarrays. Binding of reference strains and three clinical isolates of *A*. *muciniphila* and *Desulfovibrio* spp. to purified mucin was investigated.

**Results:**

Both *A*. *muciniphila* and *Desulfovibro* spp. bound to mucin. The reference strain and all clinical isolates of *A*. *muciniphila* showed increased binding capacity for UC mucin (p < .005). The *Desulfovibrio* reference strain showed increased affinity for UC mucin. The mucin binding profiles of clinical isolates of *Desulfovibrio* spp. were specific to each isolate. Two isolates showed no difference in binding. One UC isolate bound with increased affinity to UC mucin (p < .005).

**Conclusion:**

These preliminary data suggest that differences exist in the mucin binding capacity of isolates of *A*. *muciniphila* and *Desulfovibrio* spp. This study highlights the mucin microarray platform as a means of studying the ability of bacteria to interact with colonic mucin in health and disease.

## Introduction

The mucus gel layer (MGL) forms a protective barrier between colonic epithelium and colonic contents, preventing entry of bacteria while allowing diffusion of essential nutrients [1]. Mucins constitute the functional units of the MGL, with MUC2 representing the principal component of colonic mucus [2, 3]. Due to a high degree of glycosylation, mucins provide an energy source and a growth medium for mucus-associated microbiota [2], that in health exist in a symbiotic relationship with the host [4]. Due to their close proximity to the epithelium, microbes present within the MGL of the colon exert a greater effect on the host than luminal microbes [5]. The intestinal microbiota modulates a variety of host responses, including those related to metabolism which the host has not developed for itself [4, 6, 7]. Through foraging of carbohydrates from both dietary sources and colonic mucins, the microbiota provides an energy source through the production of short chain fatty acids (SCFAs) [7]. Interaction between the microbiota and colonic mucins warrants investigation, as this represents the true host microbial interface in the colon.

In ulcerative colitis (UC), changes occur in the MGL that may alter its protective capacity [8–10]. These include physical changes in the mucus barrier [11], altered mucin gene expression [12] and biochemical changes affecting the mucins [13]. Increased microbial colonisation of the MGL has been reported in UC [14]. Changes in colonic mucus and mucin may influence bacterial colonisation in the inflamed colon, due to altered availability of mucin-derived substrate, leading to an altered microenvironment. In addition there is substantial evidence of a dysbiosis in UC [15–17]. As a result, changes in relative abundances of certain bacterial taxa in the UC setting may affect mucin production and secretion [18].

This study focuses on two colonic commensals *A*. *muciniphila* and *Desulfovibrio* spp., both of which have the potential to metabolise colonic mucin. *A*. *muciniphila* is known to have mucolytic potential *in-vitro* [19] and may have a role in stimulation of the immune system and maintenance of tolerance to commensal microbes [20, 21]. *A*. *muciniphila* may also be involved in a “positive feedback loop” whereby mucolytic properties may stimulate mucus renewal [22]. Furthermore, this microbe binds to colonic cell lines and may contribute to maintenance of the integrity of the colonic epithelial cell layer [23]. In UC, the abundance of *A*. *muciniphila* is reduced [24, 25]. However, it remains to be determined whether this change is related to altered binding to colonic mucin.

*Desulfovibrio* spp. may contribute to mucosal inflammation in UC through production of potentially toxic hydrogen sulfide, released as a by-product of metabolism of sulfated mucin [26–31]. It is not known whether *Desulfovibrio* spp. is capable of directly binding mucin or whether it metabolises sulfate from mucin that has been cleaved by other bacteria.

The present study utilises a mucin microarray platform as a means of testing the hypothesis that mucolytic microbes bind to human colonic mucin and investigates the affinity for mucin in health and UC.

## Materials and Methods

### Ethical approval, patient recruitment and sample collection

Ethical approval was obtained from St. Vincent’s University Hospital Ethics and Medical Research Committee. All individuals gave informed, written consent prior to the procedure.

For the collection of mucin specimens, seven control patients undergoing colonic resection for cancer and five patients with UC undergoing colectomy were recruited. For the control mucin, paired biopsies of approximately 2 cm^2^ of mucosal tissue were resected from the excised colon at least 5 cm from the tumour. Similar paired biopsies of mucosa from patients with UC were obtained from the caecum, transverse, left colon and rectum of fresh surgical resection specimens. Patients had not received bowel preparation prior to undergoing surgery. In each case, one of the paired samples for mucin isolation was freshly frozen and one for histological analysis was stored in formalin.

Mucus was harvested and mucin purified as previously described [32, 33]. In brief, mucus was suspended in guanidine hydrochloride (final concentration 4M) to form a solution. Samples were reduced with dithiothreitol (DTT) (Sigma Aldrich) at a final concentration of 0.01M at 37°C for 5 hours and were alkylated with iodoacetamide (0.025M) (Sigma Aldrich). Mucin was purified by CsCl density gradient separation and size exclusion chromatography.

For bacterial isolation, mucosal biopsies were obtained from one control patient, and three patients with active UC. The healthy volunteer was asymptomatic and undergoing a screening colonoscopy for family history of colorectal carcinoma. This patient had no mucosal evidence of pathology. Bowel preparation was sodium picosulfate based. Exclusion criteria included: antibiotic usage or hospital admission in the six weeks prior to colonoscopy, a history of bleeding per rectum, personal history of irritable bowel syndrome or colorectal carcinoma. The biopsy was obtained using a RadialJaw 3 biopsy forceps (Boston Scientific, Natick, MA, USA) and was retrieved with a sterile needle to prevent external contamination. Biopsies from patients with UC were obtained from rectal mucosa at the time of surgical resection for disease refractory to medical management. Approximately 1 cm^2^ of mucosa was resected using sterile instruments.

### Bacterial strains, bacterial isolations and culture

The *A*. *muciniphila* reference strain ATCCBAA-835 (American Type Culture Collection, Manassas, VA) and *Desulfovibrio desulfuricans* reference strain ATCC 27774 (American Type Culture Collection) were cultured according to the suppliers guidelines using BHI (Sigma Aldrich, Dublin, Ireland) and a modified Postgate’s medium respectively. Modified Postgate’s medium was prepared as follows: K_2_PO_4_ 0.5g/L, NH_4_CL 0.5 g/L, CaSO_4_ 1 g/L, MgSO_4_.7H_2_O 2 g/L, sodium lactate 3.5 g/L, yeast extract 1 g/L, 30 g/L fastidious anaerobic broth (Lab M Ltd., Bury, Manchester, UK), and ascorbic acid 0.1 g/L and autoclaving at 121°C for 15 min. Filter sterilised FeSO_4_ was added to the modified Postgate’s medium at a concentration of 0.5 g/L immediately prior to use. All reagents were sourced from Sigma Aldrich unless otherwise stated. Cultures were placed in a shaking incubator at 200 rpm at 37°C for 16 hours under anaerobic conditions achieved by the use of AnaeroGen anaerobic gas packs (Oxoid, Basingstoke, UK).

Fresh colonic mucosal samples were placed directly into 5 ml of sterile phosphate buffered saline (PBS) immediately after resection and stored at 4°C until culturing. Immediately prior to culturing, samples were vortexed for 30 seconds and 1 ml of the PBS solution was inoculated into 50 ml of Brain Heart Infusion(BHI) broth and 50 ml of modified Postgate’s medium broth for isolation of *A*. *muciniphila* and *Desulfovibrio* spp. respectively. Cultures of *A*. *muciniphila* were incubated for 16 hours and *Desulfovibrio* spp. for up to 72 hours. Following growth, as evidenced by a cloudy appearance of BHI broth and a black precipitate accompanied by the odour of hydrogen sulfide in the case of modified Postgate’s medium, the cultures were sub-cultured onto BHI and a modified Postgate’s medium agar. The modified Postgate’s medium agar was prepared as described above plus the addition of 15 g/L of bacteriologic agar. Cultures were incubated at 37°C overnight under anaerobic conditions achieved by the use of AnaeroGen anaerobic gas packs (Oxoid). Sub-culturing was repeated until a pure growth of each isolate was obtained.

Growth of *A*. *muciniphila* was characterised by white colonies measuring approximately 0.7 mm in diameter as previously described [19]. Gram staining was performed to confirm the presence of gram negative oval-shaped cells characteristic of *A*. *muciniphila*. Colonies of *Desulfovibrio* spp. were identified as described above. Isolates of *A*. *muciniphila* and *Desulfovibrio* spp. were stored at -80°C on cryopreservative beads (MicroBank, ProLab Diagnostics, ON, Canada) until further analysis.

To confirm the identity of clinical isolates, PCR using an assay specific for each bacterial target was performed. DNA extraction was performed on a single colony from each culture by re-suspending in PBS followed by four heat/freeze cycles at 100°C and -80°C. Conventional PCR targeting the 16S rRNA gene of each target was performed using oligonucleotide primers targeting *A*. *muciniphila* (forward primer 5’- CAGCACGTGAAGGTGGGGAC– 3’ reverse primer 5’- CCTTGCGGTTGGCTTCAGAT-3’) [24] and *Desulfovibrio* spp. (forward primer 5’- CCGTAGATATCTGGAGGAACATCAG -3’, reverse primer 5’-ACARCTAGCATCCATCGTTTACAGC-3’) [34] respectively. All PCR reactions contained 1X My Taq Red Mix (Bioline, London, UK), forward primer and reverse primer at a final concentration of 200 nM. For *Desulfovibrio* spp., each reaction contained 5 μl of DNA template and for *A*. *muciniphila* reactions contained 10 μl of DNA template. Each assay run incorporated a negative control and a reference sample of cloned 16S rRNA gene from *Desulfovibrio* spp. or *A*. *muciniphila* as a positive control. All reactions were carried out on a Multigene thermocycler (Labnet International Inc., Woolbridge, NJ, U.S.A.) under the following cycling conditions: *A*. *muciniphila* 95°C for 10 seconds initially, followed by 30 cycles of 95°C for one minute, 50°C for one minute, 68°C for 10 seconds. PCR conditions for *Desulfovibrio* spp. were: 95°C for 5 minutes initially, followed by 35 cycles of 95°C for 1 minute, 62°C for 1 minute, 72°C for 45 seconds and a final extension step at 72°C for 5 minutes. PCR products were analysed by electrophoresis in a 2% agarose gel stained with 0.5 μg/ml ethidium bromide (Sigma) and visualised under ultra violet light immediately after electrophoresis. Products of *A*. *muciniphila* and *Desulfovibrio* spp. were visualised at 327 and 135 base pairs, respectively.

### Histological analysis of specimens from which mucin was isolated

Formalin fixed, paraffin embedded mucosal biopsy specimens for each mucin sample were stained using Haematoxylin and eosin stain (H&E) and High Iron Diamine-Alcian Blue (HID-AB) staining to quantify degree of inflammation and percentage sulfation as previously described [35]. For each specimen, the quantity of sulfated mucin was determined and results expressed as the percentage relative to the total mucin content for a given specimen. For histological analysis, UC specimens were scored as mild, moderate or severe inflammation, according to the system described by Geboes *et al* [36]. Control specimens were described as normal mucosa.

### Interrogation of mucin array for bacterial binding

Printing of the purified human colonic mucins on Nexterion slide H microarray slides was optimised and printed as previously described (S1 Table) [32]. Each microarray slide was printed with eight replicate subarrays, with each mucin printed in six replicates (per subarray). Microarray slides were blocked and washed as previously described [32]. Print performance and mucin glycosylation was assessed by incubating the microarray with a panel of tetramethylrhodamine-(TRITC-) labelled lectins, (see S2 Table for lectins and their incubation concentration) diluted in low salt Tris buffered saline supplemented with Ca^2+^ and Mg^2+^ ions (TBS; 20 mM Tris-HCl, 100 mM NaCl, 1 mM CaCl_2_, 1 mM MgCl_2_, pH 7.2) with 0.05% Tween-20 (TBS-T) as previously described [32].

Prior to incubation on the microarrays, *Desulfovibrio* spp. were cultured overnight in iron-free modified Postgate’s medium. Bacterial strains of *A*. *muciniphila* and *Desulfovibrio* spp. were re-suspended to an optical density 600 nm (OD_600_) of 0.1. Bacteria were cultured for 2.5 hours as described above and harvested by centrifugation of 1 ml of each culture at 16,200 x g for 1 min, the supernatant discarded and the pellet re-suspended to an OD_600_ of 1.0 in TBS. Bacterial cultures were labelled with SYTO82 nucleic acid fluorescent dye (Life Technologies, Carlsbad, CA, U.S.A.) at a final concentration of 20 μM, protected from light and incubated for 45 min at room temperature. Bacteria were washed seven times with 1 ml of TBS for each wash to completely remove unbound dye [37]. The final pellet was re-suspended to an OD_600_ of 0.5 with TBS-T.

**Fig 1:**
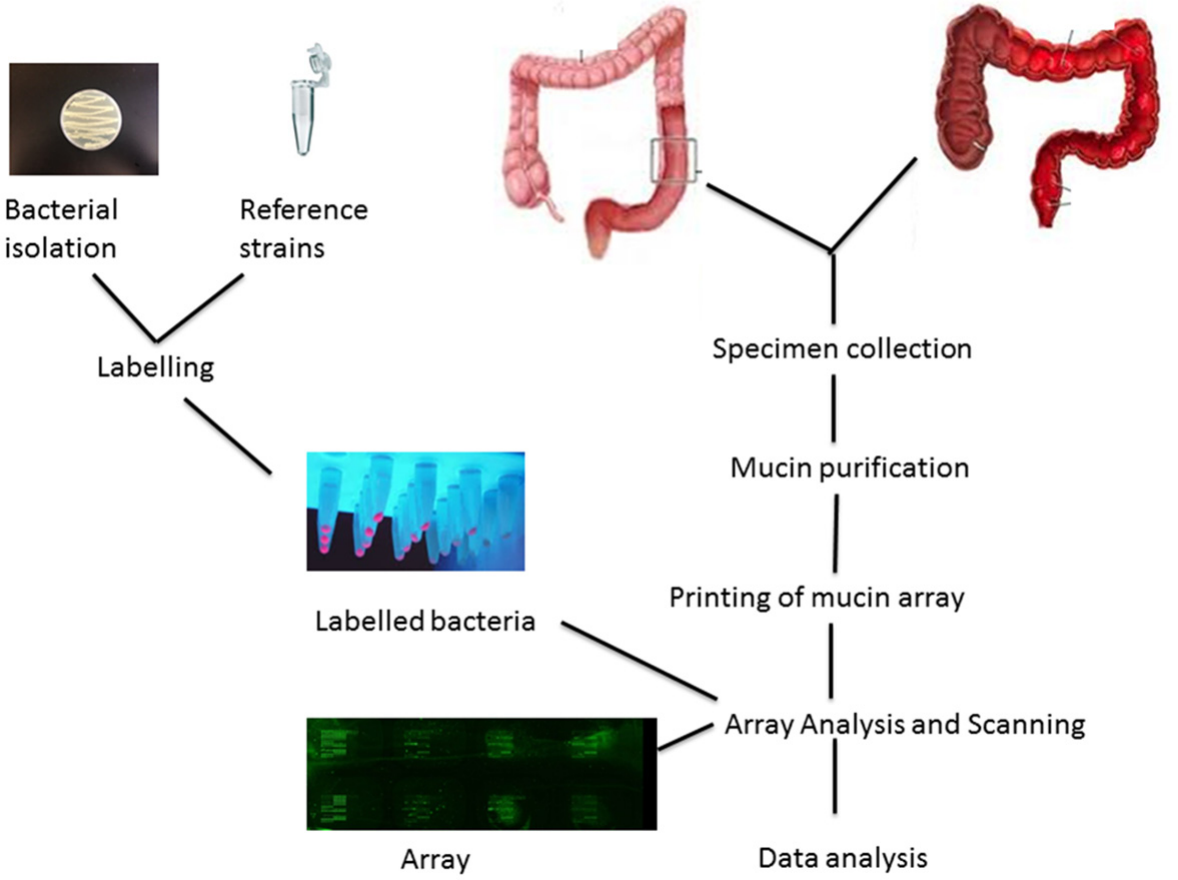
Summary of Materials and Methods.

The mucin microarray slides were initially rehydrated by incubating 70 μl of TBS per subarray using an Agilent eight-well gasket slide and incubation cassette system (Agilent Technologies, Cork, Ireland) at 37°C for 45 min. The TBS was removed and the microarray slides were subsequently incubated with 70 μl of fluorescently labelled bacteria at an OD_600_ of O.5 per subarray. Two subarrays on each microarray slide were incubated with TRITC-labelled lectins *Artocarpus integrifolia* (AIA, 15 ug/mL TBS-T, final concentration) and *Maackia amurensis* agglutinin (MAA, 10 ug/mL TBS-T, final concentration) (EY Laboratories Ltd., San Mateo, CA, USA) to monitor print performance. The slides were incubated in a shaking incubator at 200 rpm at 37°C for 1hr followed by washing five times in TBS-T, once in TBS and once in water. Slides were dried by centrifugation at 266 x g for 5 min and scanned immediately in a GenePix 4000b microarray scanner (Molecular Devices, Wokingham, UK) with the 532 nm laser using the following settings; laser power 100%, 10μm resolution and 70% PMT [33]. Analysis of each bacterial isolate consisted of two technical replicates per microarray slide and three biological replicates on different microarray slides, resulting in a total of six data sets for each isolate. These methods are summarised in Fig 1.

### Data extraction and analysis

Data were extracted from the scanned images using GenePix pro software (Molecular Devices) and then exported to Excel for subsequent analysis and normalisation as previously described [33, 37]. The median of six replicate features per subarray was handled as a single data point for graphical and statistical analysis. Data intensities across the three replicate microarray slides were normalised against the ratio of total subarray fluorescence/mean total fluorescence of the six replicate subarrays to account for inter-microarray slide variability and minimise the effect of variation in mucin printing. Mucin micro-array data are available in the supplementay information (S3 and S4 Tables)

### Glycosylation Profiles of Human Colonic Mucins

**Fig 2:**
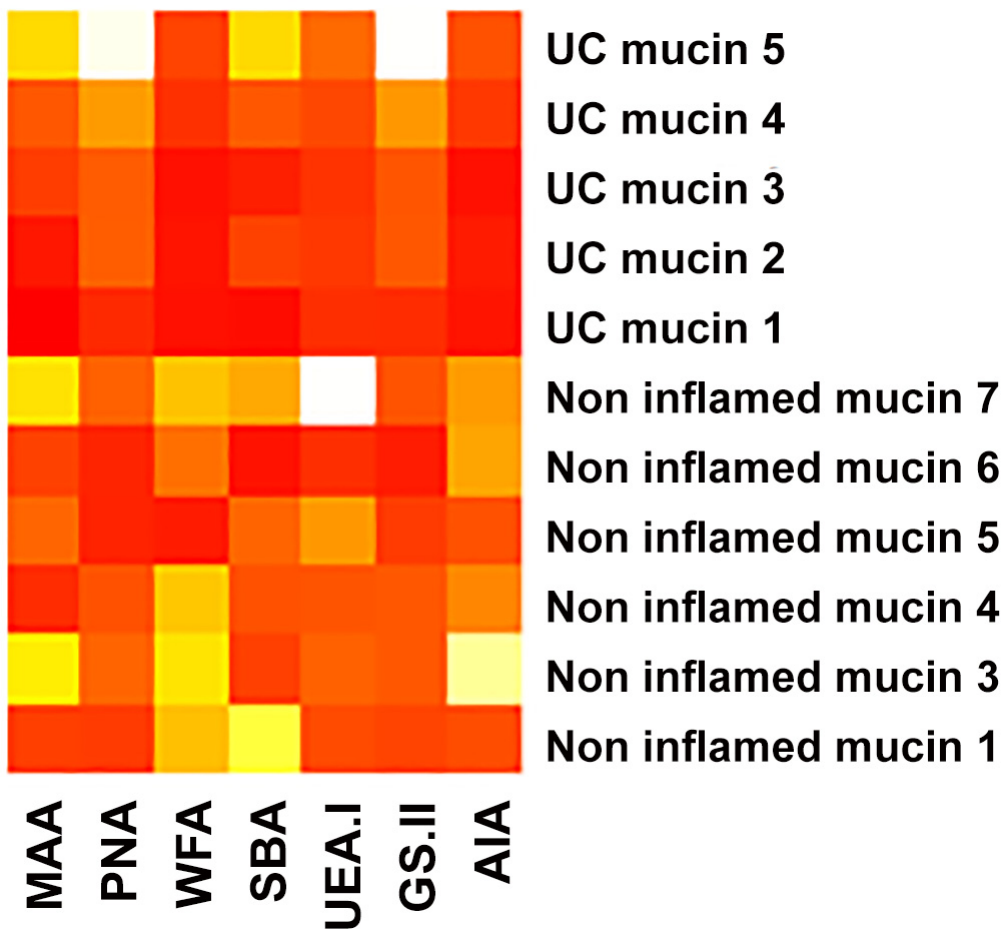
Comparison of the glycosylation profiles of human colonic mucins generated from lectin profiling of the mucin microarray. The maximum binding for colonic mucins is 8,335 RFU. The highest intensity binding is represented by red, followed by orange, yellow and white.

Data generated from lectin profiling of the mucin microarray were used to compare the glycosylation profiles of human colonic mucins in health and UC (Fig 2). Sialylation, as indicated by MAA binding, is present in the colonic mucins. Sulfation, as determined by WFA binding with low or without concomitant SBA binding, was most present in the control mucins. Varying quantities of sialylated or sulfated *O*-linked oligosaccharides were present throughout the samples as determined by varying intensities of AIA binding.

### Statistical analysis

Normalised data were exported to SPSS statistics, version 20.0 (SPSS statistics, IBM, London, U.K.) for statistical analysis. Statistical comparisons were performed based on Mann-Whitney U test and Kruskal-Wallis comparisons.

## Results

HID-AB analysis of biopsies collected from each individual indicated that UC mucin had a median percentage sulfomucin of 39.51% (IQR 32.68%) and controls a median of 57.69% (IQR 16.74%). On histological analysis of mucosal biopsies obtained from patients with UC, four were classified as severely inflamed and one as moderately inflamed according to the Geboes scoring system [36]. Control biopsies were described as normal mucosa.

Three isolates of both *A*. *muciniphila* and *Desulfovibrio* spp. were successfully cultured from different individuals. One isolate of *A*. *muciniphila* and *Desulfovibrio* spp. was isolated from a control patient, and two isolates of both species were isolated from three individuals with UC.

### *A*. *muciniphila* and *Desulfovibrio* spp. bound to colonic mucin in health and UC

**Fig 3:**
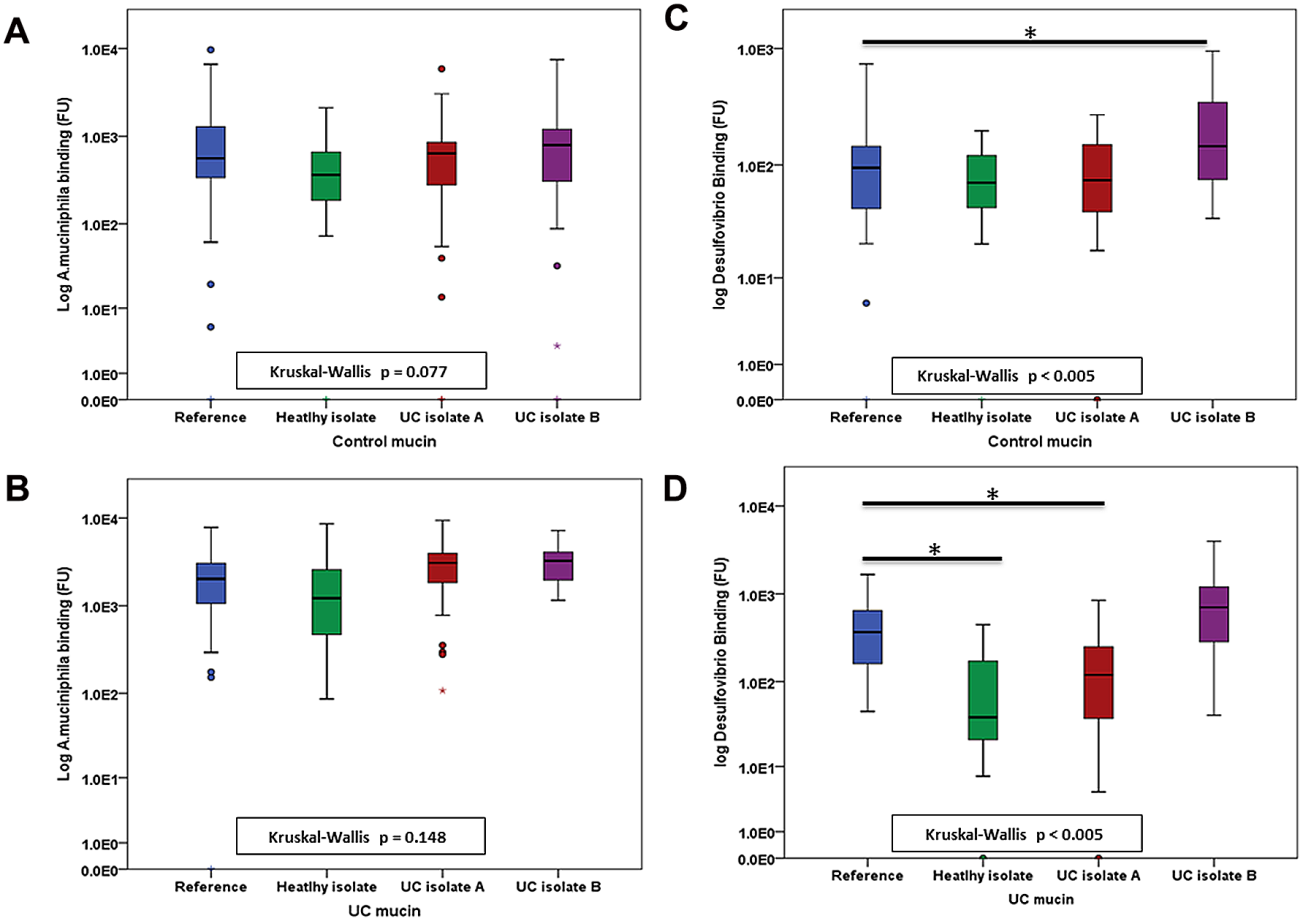
Boxplots illustrating the binding of clinical isolates and reference strains to mucin from different sources. Median binding of isolates of *A*. *muciniphila* to control mucin (a) Median binding of isolates of *A*. *muciniphila* to UC mucin (b) Median binding of isolates of *Desulfovibrio* spp. to control mucin (c) Median binding of isolates of *Desulfovibrio* spp. to UC mucin (d).

Both *A*. *muciniphila* and *Desulfovibro* bound to colonic mucin (Table 1). Clinical isolates of *A*. *muciniphila* did not differ from the reference strain with regard to binding to control and UC mucin (Fig 3a and 3b, Table 1). However, all clinical isolates of *Desulfovibrio* spp. showed differences in comparison to the reference strain *D*. *desulfuricans*. One isolate of *Desulfovibrio* spp., cultured from a patient with UC, (UC isolate B) displayed increased binding to mucin from controls compared to the reference strain (Fig 3c, Table 1), while the healthy isolate and one UC isolate (UC isolate A) of *Desulfovibrio* spp. bound to mucin isolated from UC colon with reduced affinity compared to the reference strain (Fig 3d, Table 1).

**Table 1:** Median binding values of each isolate to mucin from controls and the UC colon and Kruskal-Wallis tests comparing the binding of clinical isolates to that of the reference strain for *A*. *muciniphila* and *Desulfovibrio* spp.

*A. muciniphila*	Median binding to Control mucin(FU)	IQR	Kruskal-Wallis and Wilcoxin-Mann-Whitney	Fold change in binding compared to the reference strain	Median binding to UC mucin (FU)	IQR	Kruskal-Wallis and Wilcoxin-Mann-Whitney	Fold change in binding compared to the reference strain
			.077				.148	
Reference	559.83	970.89	n/a		2022.41	2076.05	n/a	
Healthy isolate	362.58	493.62	n/a	-0.65	1221.52	2176.76	n/a	-0.60
UC isolate A	636.67	593.58	n/a	+1.14	3075.48	2398.98	n/a	+1.52
UC isolate B	792.37	908.92	n/a	+1.42	3251.41	2165.19	n/a	+1.61
***Desulfovibrio* spp.**								
			<0.005				<0.005	
Reference	95.10	107.73	n/a		368.34	501.99	n/a	
Healthy isolate	70.44	79.30	1.00	-0.74	39.60	155.02	<0.005	-0.11
UC isolate A	73.94	113.35	1.00	-0.78	119.74	238.59	.014	-0.32
UC isolate B	145.38	283.64	.022	+1.53	712.41	930.06	.276	+1.93

Significant values are highlighted in bold text. FU corresponds to measure of bacterial binding in fluorescent units. IQR corresponds to inter quartile range.

### Isolates of *A*. *muciniphila* and *Desulfovibrio* spp. display increased binding to mucin from the UC colon

**Fig 4:**
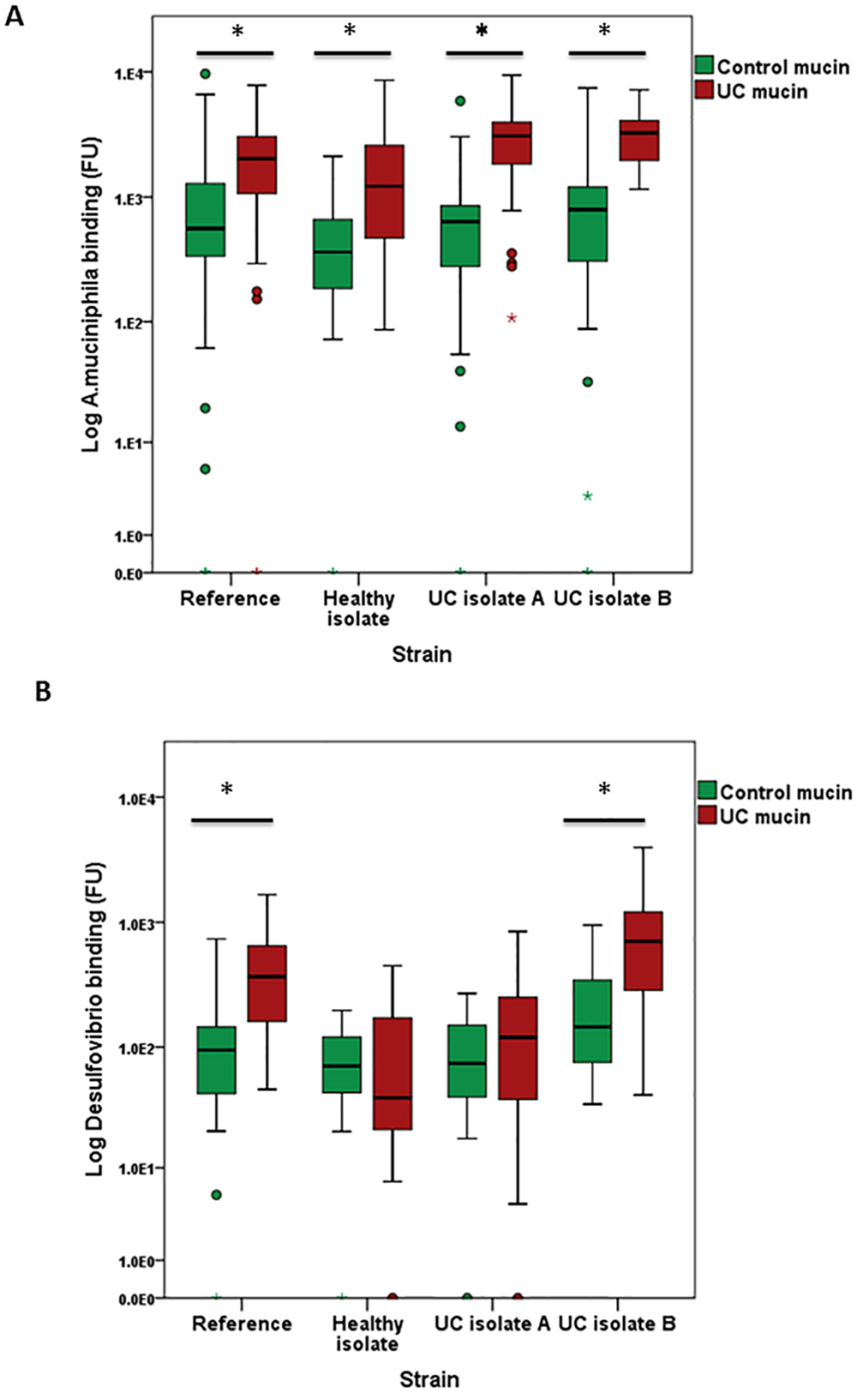
Boxplots representing the median binding of each isolate and reference strain to mucin from the UC colon compared to controls. Direct comparison of the median binding of isolates of *A*. *mucinihpila* (a). Direct comparison of median binding of isolates of *Desulfovibrio* spp. (b).

In a direct comparison of bacterial binding to mucin from UC and controls, both the reference strain and all three clinical isolates of *A*. *muciniphila* displayed increased affinity for UC mucin compared to mucin from controls (Fig 4a, Table 2).

**Table 2:** Comparison of bacterial binding to control and UC mucin as determined by the Mann-Whitney U test.

Bacterial Target	Isolate	Median Binding to mucin from controls (FU)	IQR	Median Binding to UC mucin (FU)	IQR	Mann- Whitney U(p value)
***A. muciniphila***	Reference	559.83	970.89	2022.41	2076.05	**.001**
	Healthy isolate	362.58	493.62	1221.52	2176.76	**.000**
	UC isolate A	636.67	593.58	3075.48	2398.98	**.000**
	UC isolate B	792.37	908.92	3251.41	2165.19	**.000**
***Desulfovibrio* spp.**	Reference	95.10	107.73	368.34	501.99	**.000**
	Healthy isolate	70.44	79.30	39.59	155.02	.803
	UC isolate A	73.94	113.35	119.74	238.59	.456
	UC isolate B	145.38	283.64	712.41	930.06	**.000**

Significant values are highlighted in bold text. FU corresponds to measure of bacterial binding in fluorescent units. IQR corresponds to inter quartile range.

In the case of *Desulfovibrio* spp., the reference strain and one isolate from a patient with UC (UC isolate B) bound with increased affinity to UC mucin compared to mucin from controls, while the healthy isolate and the isolate from the second patient with UC (UC isolate A) showed no difference in binding (Fig 4b, Table 2).

## Discussion

UC is associated with changes in colonic mucins thought to be related to dysregulated cross-talk between the host and an altered microbiota. Mucin binding is the first point of bacterial interaction with the host and, as such, is a key mediator of this cross-talk. The present study investigated the ability of two commensals that have been implicated in the pathogenesis of UC, to bind to colonic mucin.

These preliminary data demonstrate for the first time that both *A*. *muciniphila* and *Desulfovibrio* spp. have the ability to bind to human colonic mucin. However, no adhesins have been identified in the currently annotated genome of either *A*. *muciniphila* or *Desulfovibrio* spp. [38]. Previous studies have demonstrated the ability of *A*. *muciniphila* to degrade both porcine mucin and human mucin *in-vitro* [19, 24] and provided putative evidence that *A*. *muciniphila* express BACON (Bacteriodetes-associated carbohydrate-binding Often N-terminal), a protein believed to mediate mucin binding [38, 39]. The present results indicate the likely production of such mucin binding proteins, or the presence of carbohydrate binding motifs (CBMs), a family of domains that bind various polysaccharides, enhancing their degradation and may interact with mucin glycan structures [40–43]. Alternatively, non-specific interactions based on hydrophobicity have been described as an adhesion mechanism in the colon [44, 45].

The observed differences in binding may be explained by strain-specific differences in the binding capacity of the microbes or alterations to mucin in UC that promote increased mucin binding. The present study focused on a number of clinical isolates and their commercially available ATCC reference strain counterparts. While the identity of all isolates was confirmed by PCR, no information regarding the strain specificities was obtained. It is known that different strains of a bacterial species may possess different adhesion molecules [46–48] and that selective pressures and natural mutations have the potential to alter these adhesion molecules [49–51]. Given these observations and the selective pressure placed upon bacteria to survive in a niche environment like the colonic MGL, it is possible that strain specific differences in adhesion molecules explain the observed difference in mucin binding.

Mucin in the UC colon shows reduced sulfation [35, 52, 53], possibly the result of bacterial sulphatase activity [54, 55], as well as changes in the degree of mucin glycosylation [13]. The data presented here indicate a reduction in the median percentage sulphation in UC mucin compared to control mucin. Longman *et al*. did not report significant differences in the histochemistry of UC samples compared to healthy colorectal tissue using the same staining technique. However the study did not quantify sulfomucin content and did report reduced staining of the sulfo-Lewis mucin epitope, a finding that correlated with disease severity [10]. Alterations in mucin glycosylation patterns were also observed in the present study, as outlined in Fig 2. The resultant change in the microenvironment of the MGL may well influence the binding capacity of the resident microbiota. In health, the presence of the sulphate moiety is thought to protect mucin against degradation by colonic microbes [52]. The loss of sulphation, whether enzymatically mediated or through biosynthetic reduction, could alter the mucin substrate for bacterial binding. This may occur though the loss of a sugar binding ligand, in which case a reduction in binding would be observed. Alternatively, presentation of a new or previously cryptic ligand after desulfation, possibly mediated by enhanced susceptibility to mucinase activity [56], may result in increased bacterial binding. Exposure of such motifs could explain the increased binding of *A*. *muciniphila* and *Desulfovibrio* spp. to UC mucin.

It has previously been reported that *A*. *muciniphila* has the ability to bind to adenocarcinoma-derived cell lines Caco2 and HT-29, but not to colonic mucus [23]. Healthy colonocytes produce a mucus layer that is rich in O-acetylated sialic acids [57]. The oligo-O-acetylation of sialic acids is lost in colorectal cancer and may be an early biomarker in the adenoma-carcinoma sequence [58, 59]., a finding that may account for the high binding of *A*. *muciniphila* to adenocarcinoma-derived cell lines. Although the degree of O-acetylation of sialic acids was not evaluated in the present study, it warrants consideration as a possible important modulator of bacterial binding.

It should also be considered that the binding process itself may influence subsequent binding events. Mucin-microbe interactions have previously been investigated by Skoog *et al*., who demonstrated that weak interactions between *Helicobacter pylori* and gastric mucin result in increased expression of *H*. *pylori* adhesion factors in an *in-vitro* model [60]. It is possible that similar events may be occurring in the case of these commensals and other commensals or pathogens within the colon.

While the present study has identified differences in the affinity of *A*. *muciniphila* and *Desulfovibrio* spp. for mucin from the inflamed and non-inflamed colon, it should be acknowledged that the purified mucin used in this study lacks many of the physiological factors and constituents of mucus such as inflammatory mediators and cytokines, that *in-vivo* modulate the physiology of colonic mucus and in turn may influence microbial binding. The authors also acknowledge the low number of clinical isolates investigated in this study. While the results have indicated that strain specific differences in the binding patterns of isolates of *Desulfovibrio* spp. exist, we are hesitant to suggest that these findings reflect how healthy and UC isolates behave in all cases. Further study involving isolates from a larger number of individuals (both healthy and UC) is required to more fully understand the mediators of mucin binding *in-vivo*.

However, this work does highlight the potential for use of mucin microarray technology in the investigation of microbe-mucin binding. Manipulation of the microbiota holds great potential as a treatment modality in colorectal diseases. For such biotherapies to reach full potential, a thorough understanding of the nature of the interactions between individual bacterial species and the host is required. Assays such as those described in this study, advance knowledge of microbe-mucin interactions in UC, as well as other gastrointestinal diseases.

## Supporting Information

S1 Table Purified human colonic mucins, print information and concentrations used. (DOCX)

S2 Table Lectins used for mucin glycoprofiling, lectin specificities and concentrations used. (DOCX)

S3 Table Mucin micro-array data for *A*. *muciniphila*. (XLSX)

S4 Table Mucin micro-array data for *Desulfovibrio* spp. (XLSX)
