# Supplementary material for: A Preliminary Study Examining the Binding Capacity of Akkermansia muciniphila and Desulfovibrio spp., to Colonic Mucin in Health and Ulcerative Colitis
Source: PLoS One. 2015 Oct 22;10(10):e0135280. doi: 10.1371/journal.pone.0135280 (PMC4619660; doi:10.1371/journal.pone.0135280)
Supplement: S1 Table — (DOCX) [file pone.0135280.s001.docx]

Supporting Information

Binding capacity of *Akkermansia muciniphila* and *Desulfovibrio* spp., to colonic mucin in health and ulcerative colitis.

Helen Earley^1,2^., Grainne Lennon^1,2^., Aine Balfe^1,2^, Michelle Kilcoyne,^3,4^ Lokesh Joshi^3^, Stephen Carrington^5^, Marguerite Clyne ^1^., Sean T. Martin^2^, J. Calvin Coffey ^6^., Desmond C Winter^2^., P. Ronan O’Connell^1,2^.

**S1 Table**. Purified human colonic mucins, print information and concentrations used.

| **Sample** | **Print buffer PBS, pH 7.4 with % Tween-20** | **Mucin concentration (ug/uL)** |
| --- | --- | --- |
| Non inflamed mucin 1 | PBS 0.025% T | 0.5 |
| Non inflamed mucin 2 | PBS 0.025% T | 0.5 |
| Non inflamed mucin 3 | PBS 0.01%T | 0.5 |
| Non inflamed mucin 4 | PBS 0.025% T | 0.5 |
| Non inflamed mucin 5 | PBS 0.025% T | 0.5 |
| Non inflamed mucin 6 | PBS 0.01%T | 0.5 |
| Non inflamed mucin 7 | PBS 0.01%T | 0.5 |
| UC mucin 1 | PBS 0.025% T | 0.5 |
| UC mucin 2 | PBS 0.025% T | 0.5 |
| UC mucin 3 | PBS 0.025% T | 0.5 |
| UC mucin 4 | PBS 0.025% T | 0.5 |
| UC mucin 5 | PBS 0.025% T | 0.5 |
