# Supplementary material for: A Preliminary Study Examining the Binding Capacity of Akkermansia muciniphila and Desulfovibrio spp., to Colonic Mucin in Health and Ulcerative Colitis
Source: PLoS One. 2015 Oct 22;10(10):e0135280. doi: 10.1371/journal.pone.0135280 (PMC4619660; doi:10.1371/journal.pone.0135280)
Supplement: S2 Table — (DOCX) [file pone.0135280.s002.docx]

**S2 Table. Lectins used for mucin glycoprofiling, lectin specificities and concentrations used.**

| **Lectin** | **Abbreviation** | **Binding specificity** | **Concentration (mg/mL)** |
| --- | --- | --- | --- |
| *Ulex europaeus* agglutinin I | UEA-I | *α*-(1→2)-linked Fuc | 10 |
| *Maackia amurensis* agglutinin | MAA | Neu-*α*-(2→3)-GalNAc, Gal-3-SO_4_^2-^ > Lac | 10 |
| *Griffonia simplicifolia* lectin-II | GS-II | Terminal non-reducing α- or β-linked GlcNAc | 10 |
| Peanut agglutinin | PNA | Gal (Gal-*β*-(1→3)-GalNAc (T-antigen) > GalNAc > Lac > Gal, terminal *β*-Gal) | 15 |
| *Artocarpus integrifolia* agglutinin | AIA | Gal, Gal-*β*-(1→3)-GalNAc (T-antigen), Gal-*α*-(1→6), sialylation independent. | 15 |
| Soya bean agglutinin | SBA | GalNAc > Gal | 15 |
| *Wisteria floribunda* agglutinin | WFA | GalNAc, GalNAc-*α*-(1→6)-Gal > GalNAc-*α*-(1→3)-GalNAc (Forsmann antigen) > GalNAc >> Lac > Gal, GlcA-*α*-(1→3)-GalNAc. Also binds chondroitin sulfate. | 10 |
